# Lateral Ulnar Collateral Ligament Reconstruction Through a Mini‐Invasive Approach

**DOI:** 10.1002/atn2.70135

**Published:** 2026-06-21

**Authors:** Valeria Vismara, Nieto Carlos Murillo, Alfonso Carlos Gonzalez‐Delgado, Grégoire Ciais, Filip Cosic, Pierre Laumonerie

**Affiliations:** ^1^ Department of Orthopedic and Traumatology Università degli studi di Milano Milan Italy; ^2^ Department of Orthopedic Surgery Clinique Jouvenet Paris France; ^3^ Department of Orthopedic Surgery Clinique du Sport Paris France; ^4^ Department of Orthopedic Surgery Christus Muguerza Alta Especialidad Monterrey Mexico; ^5^ Department of Orthopedic Surgery Austin Health Heidelbeg Australia; ^6^ Department of Orthopedic Surgery Peninsula Health Frankston Australia

## Abstract

Posterolateral rotatory instability is the most common form of elbow instability, secondary to valgus, supination, and axial loading in elbow extension. The lateral ulnar collateral ligament (LUCL) is the primary restraint to posterolateral rotatory instability and multiple techniques of repair and reconstruction of the LUCL have been reported in the literature with variable outcomes. The aim of this technical note is to describe a minimally‐invasive technique using a tendon graft to perform a reconstruction of the LUCL using knotless suture anchors in an onlay fashion, without violating both the articular capsule and the common extensor origin.

## Graphical abstract

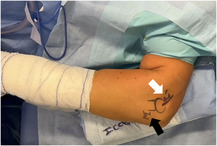

**VIDEO 1:** This technique video shows a mini‐invasive reconstruction of the lateral ulnar collateral ligament of the elbow using a knotless onlay construct. The procedure is performed with the patient in the supine position and the arm on a hand table, allowing fluoroscopic control throughout the case. The video details key anatomic landmarks and strategies to minimize soft‐tissue disruption, focusing on repairing the LCL complex, through a mini‐open approach, using knotless suture anchors in an onlay fashion. Video content can be viewed at https://doi.org/10.1002/atn2.70135.

Posterolateral rotatory instability is the most common form of elbow instability, initially described by O’Driscoll.^1^ It typically occurs secondary to valgus, supination, and axial loading in elbow extension resulting in a progressive failure of the lateral collateral ligament complex, anterior capsule, and eventually the medial collateral ligament.^2^ The lateral ulnar collateral ligament (LUCL) is the primary restraint to posterolateral rotatory instability and multiple techniques of repair and reconstruction of the LUCL have been reported in the literature, using a variety of grafts and fixation devices, with variable outcomes.^3^, ^4^, ^5^, ^6^, ^7^, ^8^ These have included the use of transosseous fixation, docking, and suture anchors, with a heterogeneous group of autografts and allografts including triceps, palmaris longus, and gracilis autografts, and Achilles allografts.^3^, ^4^, ^5^, ^6^, ^7^, ^8^ Although reconstruction has shown improved return to activity and lower complication rates than that of repair, it has also been associated with reduced range of motion compared with repair.^9^ Prior studies have described an anconeus sparing approach, avoiding anconeus detachment given its role as a secondary elbow stabilizer, and shown good clinical results, patient satisfaction, and low rates of residual or recurrent instability.^10^ The aim of this technical note is to describe a minimally‐invasive technique using a tendon graft to perform a reconstruction of the LUCL using knotless suture anchors in an onlay fashion, without violating the articular capsule or the common extensor origin.

## SURGICAL TECHNIQUE

### Positioning and Testing

**FIGURE 1:**
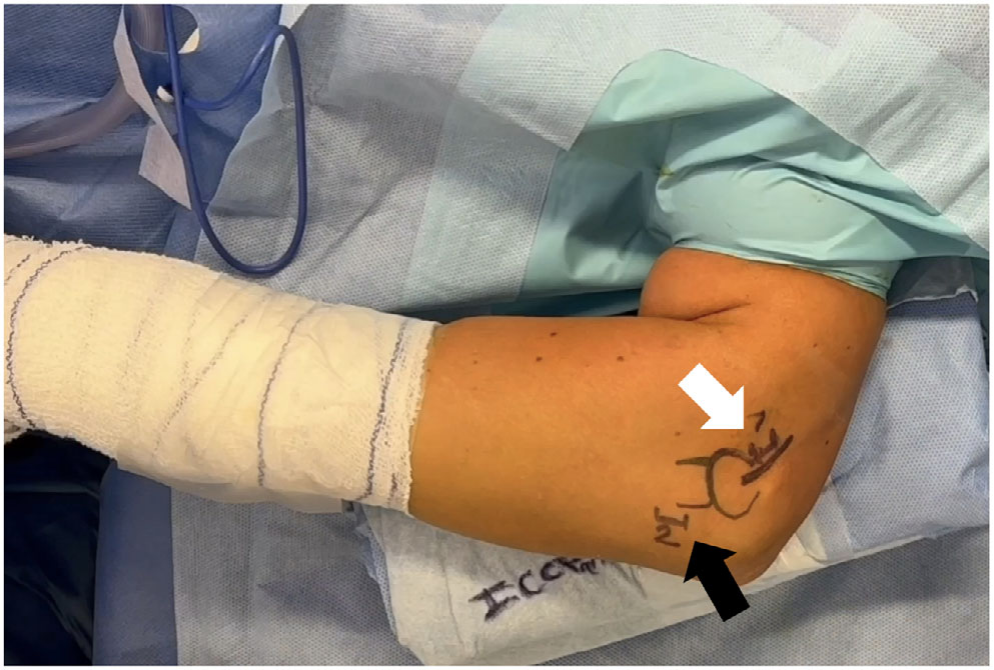
The patient is positioned supine with the left arm on a hand table. The shoulder is internally rotated and abducted to 70°, and the elbow flexed to 90° to optimize lateral elbow exposure. A pad beneath the elbow improves posterolateral access. The lateral humeral column, lateral epicondyle, and radial head are marked. The superior (white arrow) and inferior (black arrow) incisions are also highlighted.

The patient is placed supine with the arm on a hand table. After sterile preparation and pneumatic tourniquet inflation, the shoulder is internally rotated and abducted to 70°, and the elbow is flexed to 90° to allow proper visualization of the lateral side of the elbow joint. A soft pad beneath the elbow elevates the joint and improves posterolateral access (Figure 1, Video 1).

### Lateral Exposure

**FIGURE 2:**
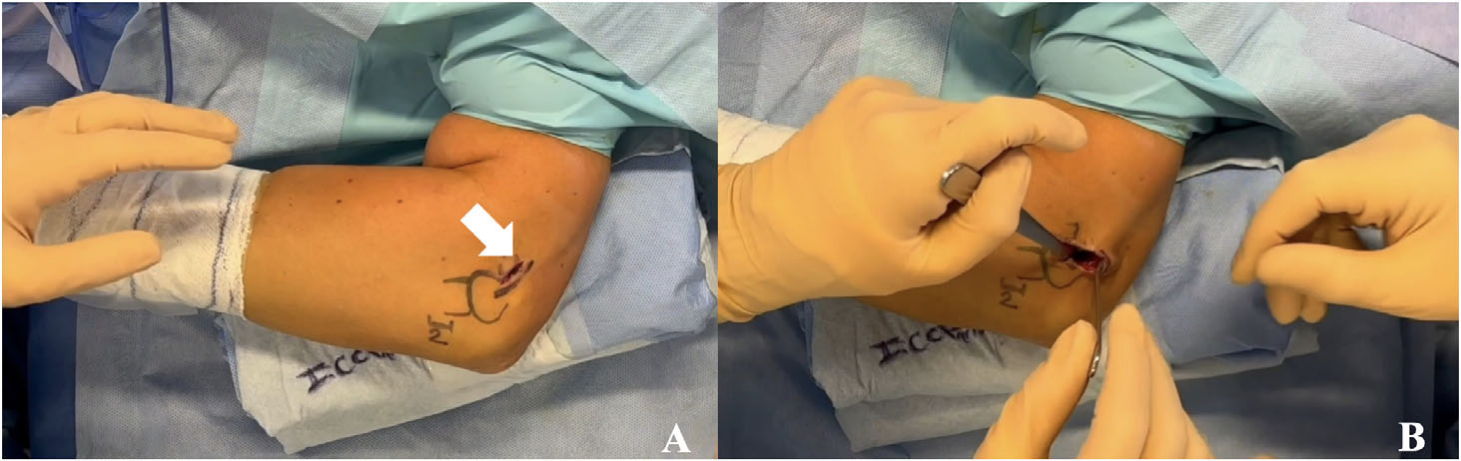
Left elbow: (A) a 1‐cm longitudinal incision (white arrow) is made at the most distal part of the lateral humeral column, above the epicondylar tendons and just medial to the lateral epicondyle. (B) Hohmann retractors are placed anteriorly and posteriorly along the distal humerus to improve exposure.

The lateral humeral column, lateral epicondyle, and radial head are marked. The proximal and distal incisions are also highlighted (Figure 1). A 1‐cm longitudinal incision is made with an 11‐blade at the most distal part of the lateral humeral column, above the epicondylar tendons and just medial to the lateral epicondyle (proximal incision). Extensor carpi radialis brevis is partially incised, along its long axis, against the bone, without detaching it. Hohmann retractors are placed anteriorly and posteriorly along the distal humerus to improve exposure (Figure 2).

### Humeral and Ulnar Anchor Placement

**FIGURE 3:**
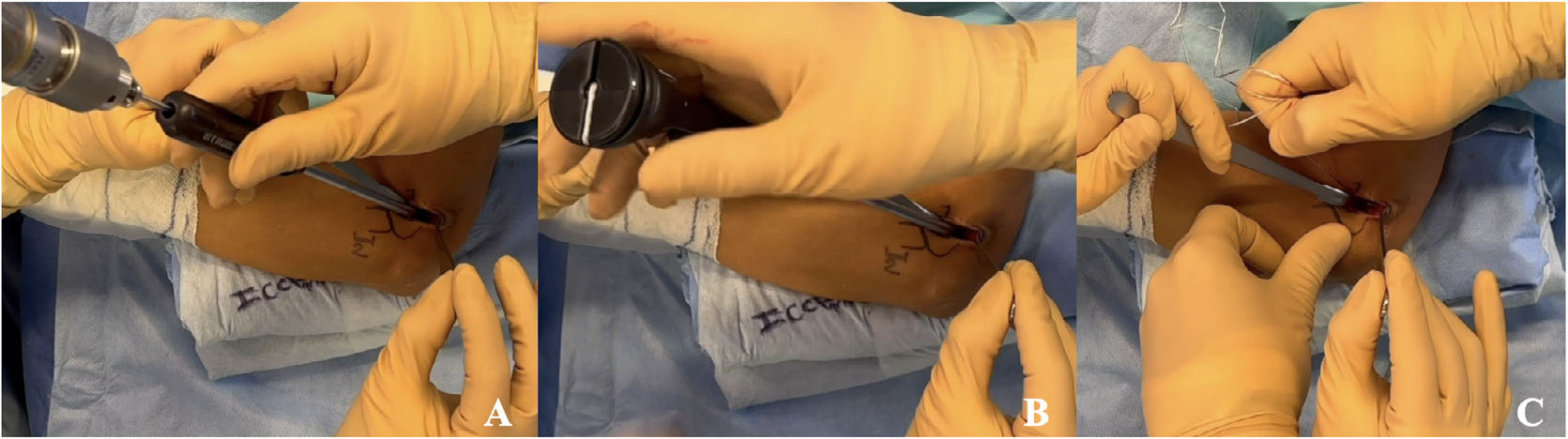
Left elbow: (A) the drilling guide is positioned at the distal aspect of the lateral humeral column, proximal to the anterior capsular insertion. (B) A 2.6‐mm FiberTak Soft Anchor is inserted at this site as the humeral fixation point of the lateral ligament complex. (C) Traction is applied to the anchor sutures to confirm secure fixation against the posterior humeral cortex.

**FIGURE 4:**
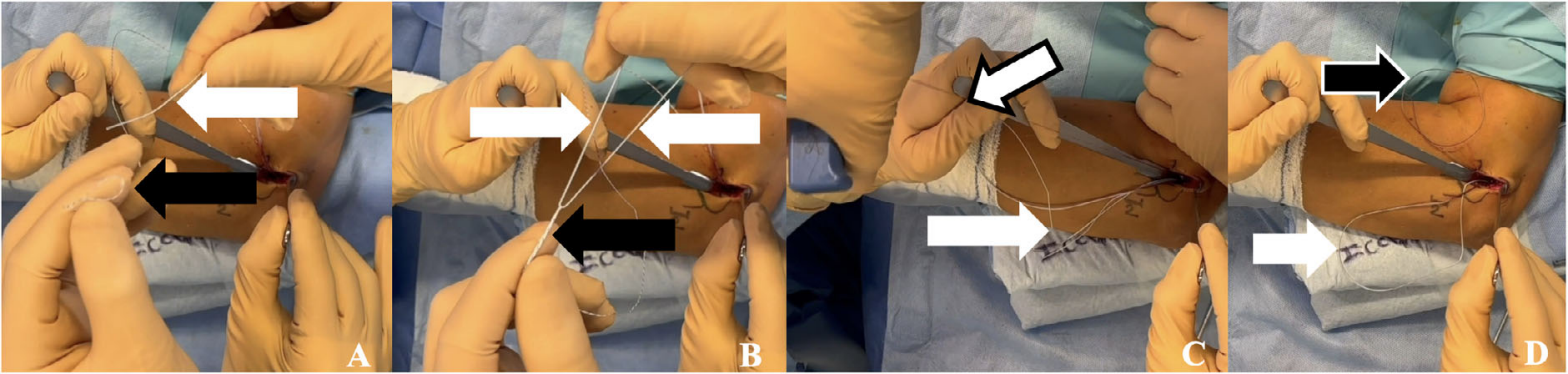
Left elbow: (A) the FiberTak anchor consists of a shuttle loop (black arrow) and a suture (white arrow). (B) The suture (white arrow) from the humeral anchor is passed through the shuttle loop (black arrow). (C) Pulling the loop draws the suture (white arrow outlined in black) into the anchor, automatically locking it and creating a knotless lasso configuration (white arrow). (D) Subsequent traction on the free suture limb (black arrow outlined in white) will tighten and definitively lock the lasso (white arrow) within the anchor.

The drilling guide is positioned at the most distal part of the lateral humeral column, and proximal to the anterior capsular insertion. A 2.6‐mm FiberTak Soft Anchor (Arthrex, Naples, FL) is inserted at this site to serve as the humeral fixation point of the lateral complex. Traction is applied to the anchor sutures to confirm secure fixation against the posterior humeral cortex. (Figure 3). The suture from the humeral anchor is passed through the shuttle loop. Pulling the loop draws the suture into the anchor, automatically locking it and creating a knotless lasso configuration. Subsequent traction on the free suture limb will tighten and definitively lock the lasso within the anchor (Figure 4).

**FIGURE 5:**
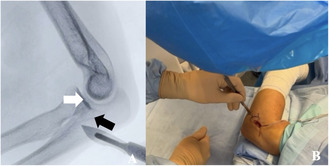
Left elbow: (A) under fluoroscopic guidance, the superior edge of the radial head (white arrow) and the supinator crest (black arrow) are identified. (B) A second skin incision is made just distal to the supinator crest of the ulna, below the superior edge of the radial head.

**FIGURE 6:**
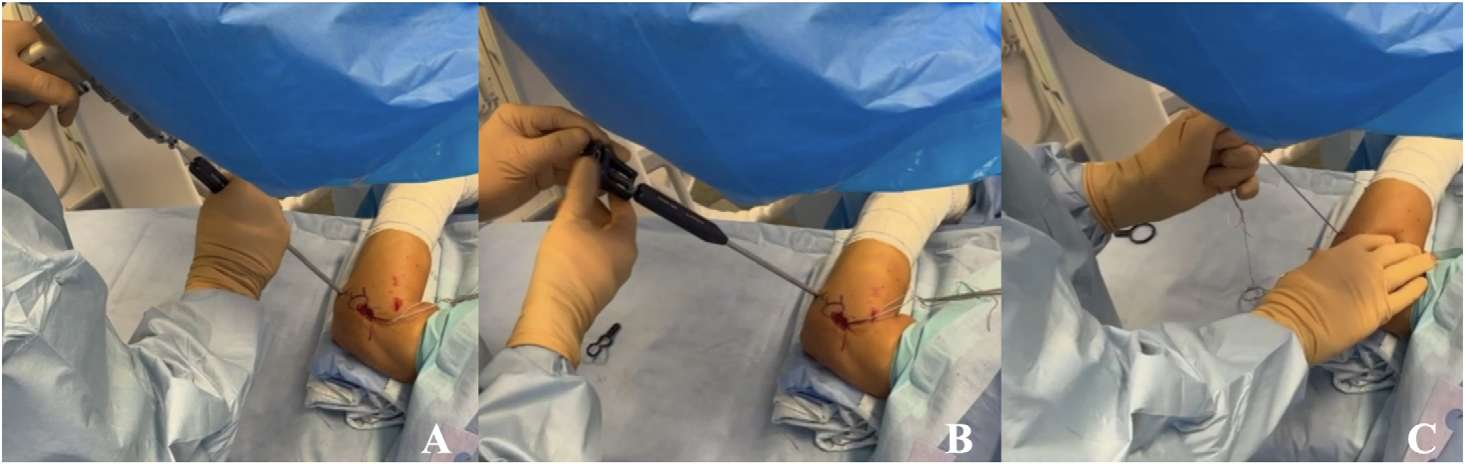
Left elbow: (A) the guidewire is positioned just beneath the supinator crest of the ulna, distal to the superior edge of the radial head. (B) A second 2.6‐mm FiberTak Soft anchor is inserted bicortically perpendicular to the cortex. (C) Traction is applied to the anchor sutures to confirm secure fixation against the medial ulnar cortex.

Under fluoroscopic guidance, a second distal skin incision (0.5 cm) is performed below the radial head, 1 cm distal to its superior edge, just distal to the supinator crest of the ulna (Figure 5). A guidewire is directed through the same incision and positioned just beneath the supinator crest of the ulna, distal to the superior edge of the radial head. A second 2.6‐mm FiberTak Soft Anchor is then placed bicortically at this location (Figure 6). Traction is applied to the anchor sutures to confirm secure fixation against the medial ulnar cortex.

### Allograft Preparation and Seating

**FIGURE 7:**
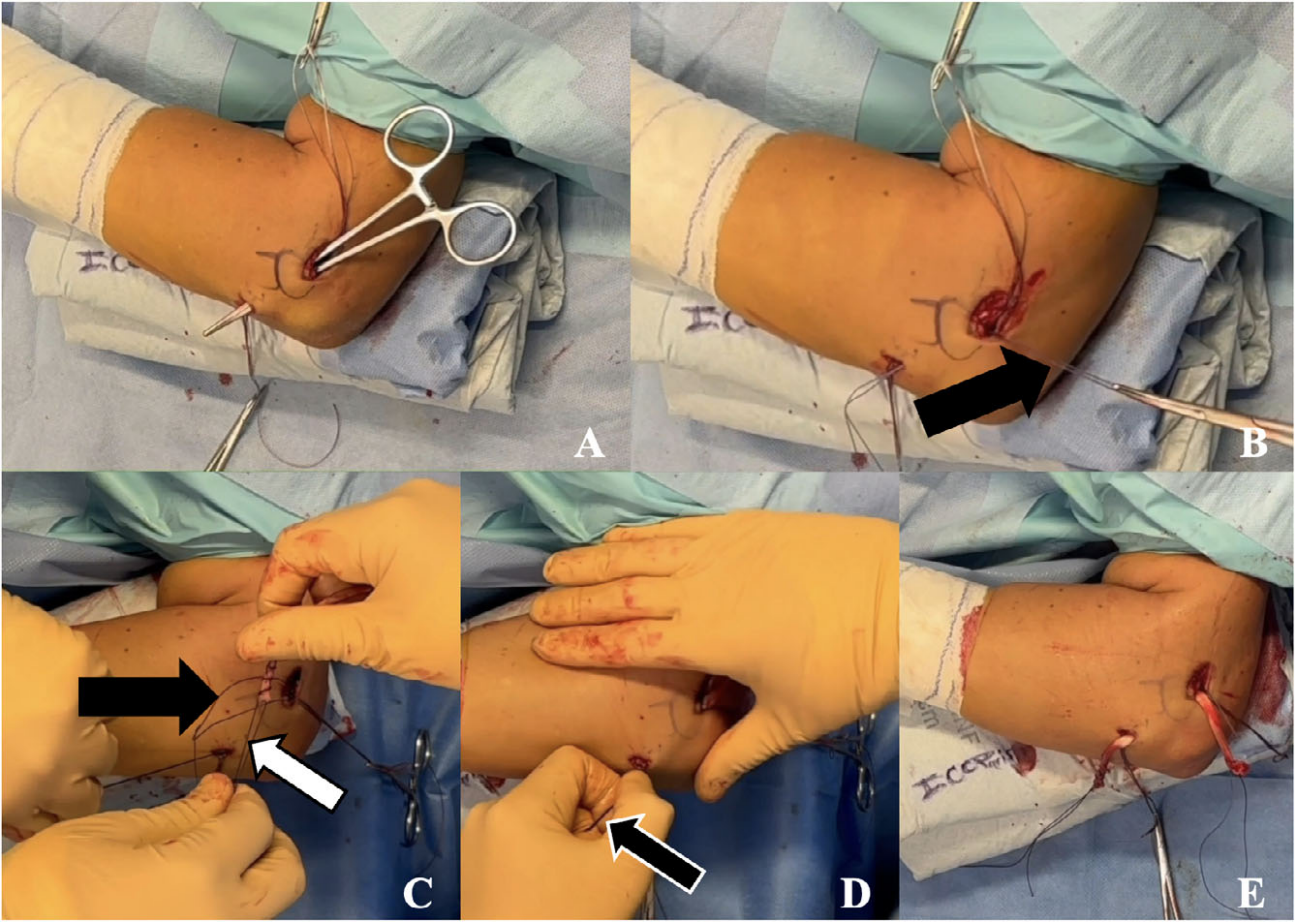
Left elbow: (A) a Kelly clamp is used to bluntly create a soft‐tissue tunnel along the LUCL pathway, between the capsuloligamentous plane and the lateral epicondylar tendons. (B) A shuttle loop (black arrow) is passed from distal to proximal through the tunnel using the Kelly clamp. (C) The gracilis graft is whip‐stitched with Krackow sutures (white arrow outlined in back), and the distal suture is passed through the shuttle loop (white arrow outlined in black). (D) Traction on the shuttle loop (black arrow outlined in white) pulls the graft from proximal to distal. (E) The graft passes through the humeral entry point, soft‐tissue tunnel, and exits distally at the ulna. (LUCL, lateral ulnar collateral ligament.)

A soft tissue tunnel, between the capsuloligamentous plane and the lateral epicondylar tendons, is created bluntly with a Kelly clamp, along the pathway of the LUCL, allowing smooth passage of the graft without constrictions. A shuttle loop is passed from distal to proximal through the tunnel using the Kelly clamp. A gracilis graft (≥12 cm long), either autograft or allograft, is whip‐stitched with Krackow sutures. The distal suture is passed through the shuttle loop. Traction on the shuttle pulls the graft from proximal to distal. The graft passes through the humeral entry point, soft‐tissue tunnel, and exits distally at the ulna (Figure 7).

### Reconstruction of LUCL

**FIGURE 8:**
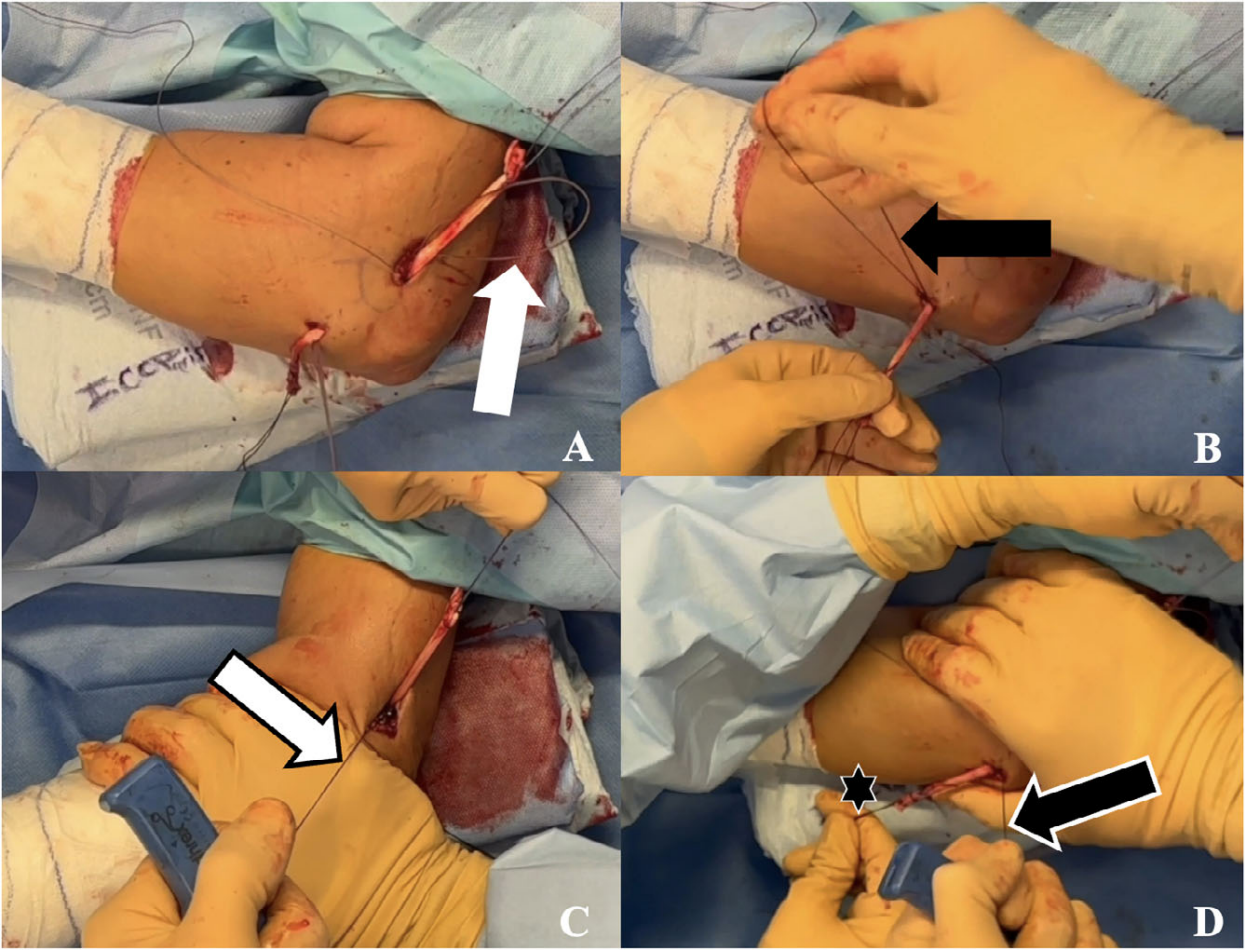
Left elbow: (A) the proximal end of the graft is passed beneath the loop (white arrow) of the proximal FiberTak Soft Anchor. (B) The distal end of the graft is passed beneath the loop (black arrow) of the distal FiberTak Soft Anchor. (C) Axial traction is applied on the humeral anchor (white arrow outlined in black), perpendicular to the lateral humeral column, to seat the graft firmly against the cortex and achieve initial humeral fixation. (D) At the ulnar anchor, with the elbow flexed at 60°, with the forearm in neutral rotation, a strong distal traction is applied along the graft axis (white arrow outlined in black), and the lasso is locked by pulling perpendicular to the ulnar cortex (black arrow outlined in white).

Once the graft has been passed, the suture from the humeral FiberTak anchor is inserted into its shuttle loop. Pulling on the loop draws the suture into the anchor, automatically locking it and creating a knotless lasso configuration. The proximal end of the graft is passed beneath the loop of the proximal FiberTak Soft Anchor. The distal end of the graft is passed beneath the loop (black arrow) of the distal FiberTak Soft Anchor. Axial traction is applied on the humeral anchor along the axis of the anchor, perpendicular to the lateral humeral column, to ensure firm fixation of the graft against the cortex. This step provides the initial humeral fixation. Fixation and graft alignment are confirmed visually and by gentle traction before proceeding to the next stage of reconstruction (Figure 8).

**FIGURE 9:**
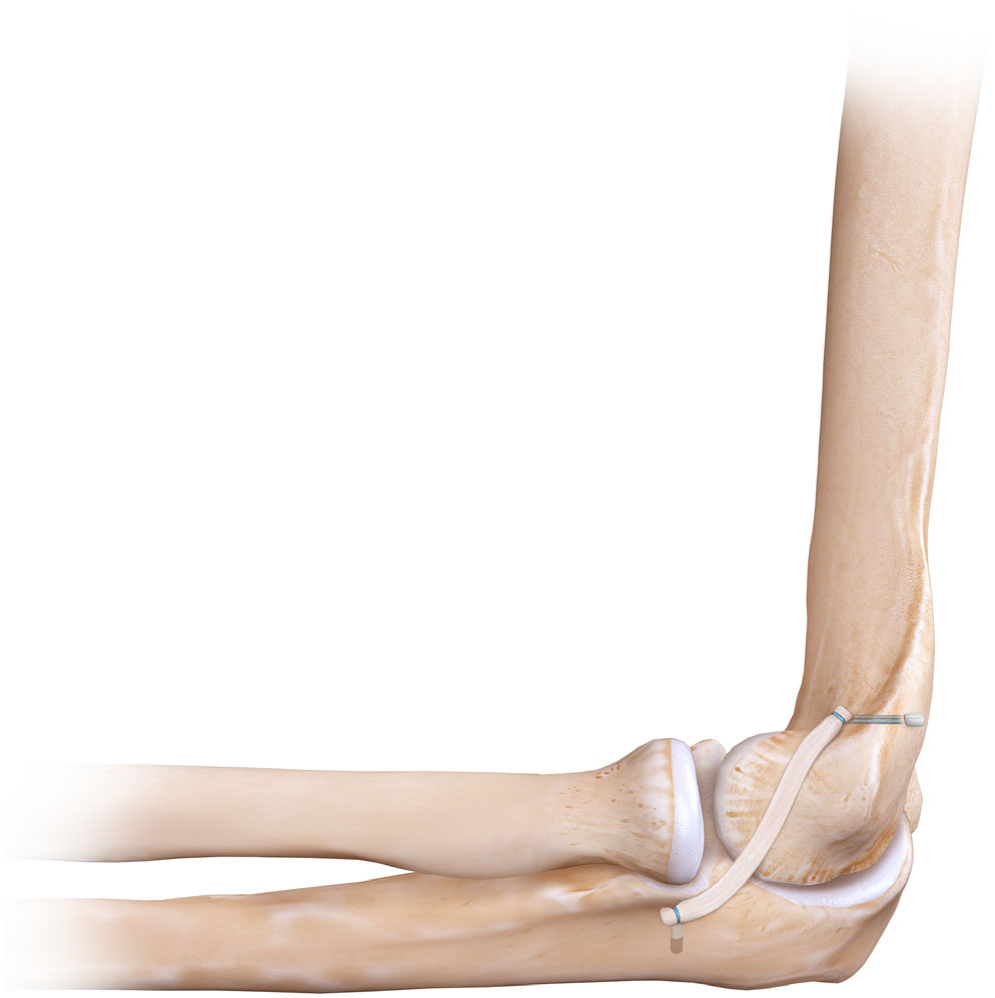
Left elbow: graphical representation of the technique in a right elbow, illustrating lateral ulnar collateral ligament reconstruction using a tendon graft and 2 soft‐tissue anchors. The graft passes between the capsuloligamentous plane and the lateral epicondylar tendons. On the humeral side, the anchor is placed bicortically through the anterior and posterior cortices. On the ulnar side, a bicortical soft‐tissue anchor is positioned just distal to the supinator crest, below the superior edge of the radial head.

At the ulnar anchor, the same step is repeated, but with the elbow flexed at 60°, with the forearm in neutral rotation: the distal end of the graft is passed beneath the loop of the distal FiberTak Soft Anchor previously placed at the LUCL insertion on the supinator crest of the ulna. Axial traction is applied on the allograft while locking the fixation to seat it firmly against the ulnar cortex (Figure 8). Both sides of the graft, proximal and distal, are trimmed and sutures are cut. The elbow is cycled through full motion to confirm stable, smooth tracking without over‐tension (Figure 9).

### Postoperative Care

Postoperative care follows the “Fix it, Move it” principle: no immobilization, immediate cryocompression therapy, and active motion, with immediate stretching and strengthening of the lateral epicondylar and triceps muscles. Shoulder forward elevation and abduction are limited for 3 weeks to prevent gravity‐induced varus stress, while avoiding passive mobilization. Return to light sports is allowed at 1.5 months, and return to high‐demand activities at 2 to 3 months.

A step‐by‐step approach and pearls and pitfalls are reported in Table 1; advantages and disadvantages of this surgical technique are reported in Table 2.

**TABLE 1:** Step‐by‐Step Technique, Pearls and Pitfalls

**Step‐by‐Step Technique**	**Pearls and Pitfalls**
Patient positioning and testing	
• The patient is positioned supine with hand table extension	• A soft pad is placed under the elbow to improve visualization of the soft tissue and allow a varus force to be applied during surgery
• Pneumatic tourniquet is applied on the upper arm	
• The upper extremity is prepared in the sterile fashion	
• Pneumatic tourniquet is insufflated at 250 mm/hg	
• The shoulder is rotated internally and abducted at 70°	
• The elbow is flexed 90°	
Lateral exposure	
• Lateral humeral column, lateral epicondyle, and radial head are marked	• Mini‐open incision of 1 cm
• A 1 cm incision is made with an 11‐blade	• The articular capsule remains untouched, as well as the CEO
• The incision is above the epicondylar tendons and medial to the lateral epicondyle	• Hohmann retractors help to visualize the bone directly
• Extensor carpi radialis brevis is partially incised, along its long axis	
• Hohmann retractors are placed anteriorly and posteriorly	
Humeral and ulnar anchor placement	
• The drilling guide is positioned perpendicular to the cortex located at the most distal part of the lateral humeral column, just proximal to the anterior capsular insertion	• It is essential to keep the guide perpendicular to the cortex and proximal to the anterior capsular insertion
• The 2.6‐mm FiberTak Soft anchor is inserted to serve as the humeral fixation point in the lateral complex	• An oblique drilling trajectory may lead to reduced anchor stability
• Under fluoroscopic guidance, an incision is performed in line with the radial head	• Fluoroscopic guidance is used for the ulna anchor placement
• The incision is posterior to the radial head	• Ulnar mini‐open incision of 1 cm
• The guidewire is positioned just beneath the supinator crest of the ulna, distal to the superior edge of the radial head	• Knotless FiberTak fixation is bicortical in the ulna
• A second 2.6‐mm FiberTak Soft anchor is inserted bicortically perpendicular to the cortex	
Allograft preparation and seating	
• A soft tissue tunnel is created bluntly along the pathway of the LUCL, between the capsulo‐ligamentous plane and the lateral epicondylar tendons	• It is essential to ensure a smooth passage of the allograft before fixing the graft
• The allograft is whip‐stitched with Krackow sutures on both sides	• A gracilis tendon graft can be used
• The graft is passed from distal (ulnar incision) to proximal (humeral incision) through the tunnel	
Reconstruction of the LUCL	
• The suture from the humeral and ulnar FiberTak Soft anchors are inserted into its shuttle loop	• Traction must be perpendicular to the axis of the anchors
• Pulling on the loops draws the sutures into the anchors, automatically locking them and creating a knotless lasso configuration	• Before fixing the distal end of the graft at the ulnar anchor, the elbow is placed at 60° of flexion with the forearm in neutral rotation
• The allograft is inserted into the humeral and ulnar lassos	
• Traction is applied along the axis of the humeral anchor, perpendicular to the lateral column of the humerus, ensuring firm fixation of the graft against the cortex	
• The elbow is flexed to 60° with the forearm in neutral rotation	
• Traction along the ulnar anchor axis, combined with axial traction on the distal edge of the allograft, allows the fixation to lock and seats the graft firmly against the ulnar cortex	
• Both proximal and distal ends of the graft are trimmed, and the sutures are cut	
• Stability of the elbow and a full range of motion is confirmed	

CEO, common extensor origin; LUCL, lateral ulnar collateral ligament.

**TABLE 2:** Advantages and Disadvantages

**Advantages**	**Disadvantages**
Minimally invasive approach	Donor site morbidity
Short operative time (30 min) with few procedural steps	Graft failure by anchor pull out
Anconeus‐ and lateral epicondylar tendon‐sparing	Graft failure due to a ‘guillotine effect’ from the lasso configuration of knotless anchors and axial traction on the graft
Low risk of cortical weakening compared with inlay construct	Lower bone‐to‐graft contact area compared with inlay constructs
Simplified graft tensioning and adjustment with onlay fixation	Non‐anatomic humeral anchor placement with respect to the native LUCL footprints
Enables early rehabilitation including immediate active motion	

LUCL, lateral ulnar collateral ligament.

## DISCUSSION

Multiple surgical techniques for the reconstruction of the LUCL have been described using a variety of tendon grafts, either autografts or allografts.^3^, ^4^, ^5^, ^6^, ^7^, ^8^ The docking technique originally described by Jones et al. in 2012 is the most common method in use in contemporary practice; however, the technique has shown recurrent instability rates as high as 25%.^3^ In contrast, our knotless, onlay technique performs a reconstruction with a tendon graft without violation of the extensor origin and soft tissue envelop, which could, in theory, allow an early mobilization and return to activity. The use of knotless anchors and an onlay technique shortens operative time, reduces the required surgical exposure, and removes the risk of tunnel osteolysis or fracture and resultant graft failure while maintaining a broad bone surface for graft incorporation.

The use of a minimally‐invasive dissection prevents iatrogenic injury to the common extensor origin, an important secondary stabilizer of the lateral elbow, and the articular capsule, allowing for earlier rehabilitation and return of range of motion, reduced postoperative pain, and reduced operative time as a repair of the common extensor origin is not required following LUCL reconstruction. Fluoroscopic guidance can help to reduce injuries to unintended structures that could be foreseen due to a limited exposure. Postoperative stiffness is a known complication of LUCL reconstruction and occurs not uncommonly.^11^ By remaining extracapsular with this technique there is no plication of the capsular structures or risk of formation of intra‐articular adhesions, theoretically reducing the risk of any postoperative loss of range of motion.^4^, ^10^

Persistent lateral epicondylar pain and tendinopathy have been reported in up to 12% of patients undergoing LUCL reconstruction secondary to iatrogenic injury to the common extensor origin.^10^, ^12^ The preservation of the common extensor origin using this technique eliminates the risk of lateral epicondylar pathology and additionally the concomitant preservation of the common extensor origin, anconeus, and triceps increases elbow stability and allows rapid rehabilitation. Prior biomechanical studies have highlighted the importance of the anconeus and triceps as secondary stabilizers of the elbow,^13^ and although anconeus sparing reconstructions have previously been described, series have continued to show recurrent instability rates up to 12.9% where the common extensor origin and triceps have been violated secondary to surgical approach and graft harvest.^4^, ^12^

Prior studies have shown minimal differences between autograft options, with gracilis and palmaris autografts both showing similar mechanical properties and clinical outcomes when used for medial collateral ligament reconstruction of the elbow.^14^, ^15^ Our preference for the use of a gracilis autograft in this technique is secondary to the anatomical reliability of the gracilis graft and the uniform presence of a gracilis in all individuals, rather than a palmaris longus autograft. If the gracilis tendon was to be wrongfully harvested, the semitendinosus tendon is harvested to provide an increased graft diameter, without the need for a second incision. Although no evidence exists regarding graft diameter and failure rates in the elbow, the reasoning in our technique is supported by literature in ACL reconstruction, clearly showing reduced rerupture rates with increased graft diameters.^16^ Nevertheless, either a gracilis autograft or allograft can be used. The use of an allograft could prevent donor site morbidity.

Overall, this technical note describes a minimally‐invasive technique using a tendon graft to perform a reconstruction of the LUCL using knotless suture anchors in an onlay fashion, without violating both the articular capsule and the common extensor origin.

## DISCLOSURES

The authors (V.V., N.C.M., A.C.G‐D., G.C., F.C., P.L.) declare that they have no known competing financial interests or personal relationships that could have appeared to influence the work reported in this article.

## ACKNOWLEDGMENTS

The surgical video and the illustrative figures accompanying this article were produced in collaboration with Arthrex (Naples, FL), which provided technical support and equipment.
